# Hepatitis C virus-related policy-making in Iran: a stakeholder and social network analysis

**DOI:** 10.1186/s12961-019-0442-1

**Published:** 2019-04-16

**Authors:** Masoud Behzadifar, Hasan Abolghasem Gorji, Aziz Rezapour, Alireza Rezvanian, Nicola Luigi Bragazzi, Soudabeh Vatankhah

**Affiliations:** 1 Health Management and Economics Research Center, Iran University of Medical Sciences, Tehran, Iran; 2 Department of Health Services Management, School of Health Management and Information Sciences, Iran University of Medical Sciences, Tehran, Iran; 3 School of Computer Science, Institute for Research in Fundamental Sciences (IPM), Tehran, Iran; 4 School of Public Health, Department of Health Sciences (DISSAL), University of Genoa, Genoa, Italy

**Keywords:** HCV, policy-making, Iran, stakeholders analysis, social network analysis

## Abstract

**Background:**

Hepatitis C virus (HCV) infection is a major public health challenge worldwide. Implementing policies to cope with this challenge requires commitment from all stakeholders at various levels, and all necessary resources should be mobilised. Support for various HCV-related stakeholders can reduce the challenges and obstacles that can be encountered during the programme implementation. The present study aims to identify all stakeholders involved with HCV-related policy-making in Iran at different steps (policy development, implementation and evaluation) and to characterise them in terms of interest, position, power and influence, in order to provide valuable information for appropriate decision-making and design. The present study can also serve as a case study for healthcare systems in other countries.

**Method:**

An approach based on social network analysis was utilised. Data collected included relevant document searches and in-depth interviews to a sample of 18 key informants.

**Results:**

Various stakeholders were found to be involved with HCV-related policies in Iran. The extent of their participation and support in policy-making varied. Specifically, international agencies had a high interest for HCV-related policy-making, whereas media and members of the private sector were characterised by a medium interest and governmental and non-governmental bodies by a highly variable interest, ranging from low to high, depending on the specific organism. Moreover, media and members of the private sector, non-governmental institutions and international agencies were rated low in terms of position, whereas governmental actors were rated low to high. Media were rated medium in terms of power, whereas international agencies and members of the private sector were respectively rated low to medium and low. Non-governmental actors were rated low, whilst governmental bodies were rated low to high. Finally, media, members of the private sector and international agencies were rated medium in terms of influence, whereas non-governmental and governmental actors were respectively rated low to medium and low to high.

**Conclusion:**

Policy-making involves trust, negotiation and integration of the different views of all stakeholders. Social network analysis was critical for identifying stakeholders and showing that, in Iran, involvement in HCV-related policy-making is generally low. This information is of practical implication for policy- and decision-makers regarding the adoption of more favourable and effective strategies.

## Background

Hepatitis C virus (HCV) infection is a major public health challenge worldwide [1]. Chronic HCV infection can potentially lead to complications such as decompensated cirrhosis, liver failure and hepatocellular carcinoma [2]. Prevention, control, diagnosis and treatment are essential and should receive special attention from health decision- and policy-makers [3]. It is estimated that, globally, approximately 71 million people are affected by chronic HCV, and approximately 399,000 people die each year due to cirrhosis and hepatocellular carcinoma [4]. WHO reported that the highest prevalence of HCV was found in the Eastern Mediterranean Regional Organization (EMRO) region, with a rate of approximately 2.3%, whilst Europe has a prevalence of approximately 1.5% [5]. Health policy- and decision-makers are working to implement HCV-related programmes, mitigating the burden generated by the infection and providing effective treatment to patients [6]. Despite the lack of funding, efforts are made to reduce the health-related costs of the illness [7] and to provide a variety of financially sustainable programmes in order to reduce mortality and transmission of disease to the general population [8].

Implementing different policies to properly cope with HCV requires commitment from all stakeholders at the international, national and regional levels, with all facilities and resources that should be effectively mobilised to implement these policies. Support for various HCV-related stakeholders can reduce problems and obstacles that can be encountered during the implementation of the programme [9]. Support can include participation in policy-making, advising and suggesting appropriate policies, providing adequate funding needed to implement policies, discussing and resolving potential disagreements over policy implementation, and engaging with policy- and decision-makers to counteract the spreading of HCV [10].

Healthcare problems are complex, nonlinear and multi-factorial, and the various stakeholders involved in the process of policy-making may have a different impact on them [11]. Stakeholders include all the individuals, groups and organisations that can influence a given health-related issue. The diversity of views among stakeholders can affect the process of policy implementation [12]. Understanding and characterising stakeholders can raise awareness of the various dimensions underlying the decision-making process and potentially improve it. Stakeholder analysis is a systematic process that consists of identifying and characterising stakeholders in terms of interests, power and relationships to a given health-related policy [12].

Concerning HCV in Iran [13], some studies have been conducted on the prevalence of this infection in different groups. For example, the prevalence rate is 0.6% in the general population [14], 0.5% in blood donors [15], 41.3% in injection drug users [16], 19% in thalassemia patients [17], 28% in prisoners [18], 11% in haemodialysis patients [19], and 2.4% in street children [20]. Based on these epidemiological findings, health policy- and decision-makers are implementing various ad hoc policies to cope with the disease [21]. However, as previously mentioned, the presence of different stakeholders with different interests and expectations towards health-related policies is one of the challenges to the implementation of various programmes [22], in that it is difficult to meet all expectations. The systematic identification and study of the stakeholders involved with a given policy through social network analysis (SNA) can enable the development of a constructive dialogue and of interactions based on their position, interests and impact on that policy [23]. Understanding the actors and their interests will make it possible to know how to develop and implement policies to counteract the spreading of HCV. Stakeholders, in relation to a given health-related policy, are actors that can have a direct or indirect effect/influence and may increase or weaken the authority and effects of that policy [24].

The Ministry of Health and Medical Education (MoHME) is the main sponsor of health in Iran. The government, according to the different needs of the ministries and their programmes, provides the necessary budget. The parliament, according to the representatives of the delegates, ultimately approves the budget and adopts measures to implement it [25]. The MoHME allocates adequate financial resources for each programme and runs them through its medical universities (MUs) in all provinces. For other programmes and policies, other ministries can support the MoHME [26]. More specifically, the National Hepatitis Committee based at the Centres for Disease Control and Prevention, focuses on major HCV-related programmes in order to implement them. HCV-related policies are run through the Secretariat of the Supreme Council for Health and Food Security, in collaboration with other ministries if needed. Implementation of HCV-related plans is under the responsibility of the MoHME, health authorities and organisms, and other organisations that independently monitor the various processes through different quality indicators [27].

The present investigation aims to analyse the roles and characteristics of all stakeholders involved in HCV-related policies in Iran in order to provide valuable information for designing proper plans and making appropriate decisions about HCV-related policies. It may also serve as a case study for healthcare systems in other countries.

## Methods

### Stakeholder identification and analysis

Based on our aim, in order to examine the various dimensions and effects that stakeholders can have on HCV-related policies in Iran, the suggestions and the theoretical framework adopted by the World Bank [28] and by Varvasovsky et al. [24] were deemed appropriate and chosen for our study. As such, stakeholders were analysed and characterised based on four items, namely interest, position, power and influence.

Data were collected in two phases. Firstly, identification of the stakeholders (through interviews and documents) and, secondly, assessment of the view of participants towards stakeholder’s roles (through expert opinions). In-depth interviews were conducted with key informants involved in HCV-related programmes and policies. The full list of participants is shown in Table 1.

**Table 1:** List of participants enrolled in the present study

Participants	No.
Policy-makers	3
Specialist doctors	2
Nurses	1
Health centre staff	1
Hospital manager	1
Insurance manager	1
Judicial system	1
State Prisons and Security and Corrective Measures Organisation	1
Pharmaceutical companies managers	1
University professors	2
Non-governmental organisations	2
Iranian Red Crescent	1
Social media	1

The main question framed was, ‘who are the main HCV-related actors (such as organisations or institutions) involved with the processes of policy-making in Iran?’ Within this step, HCV-related policy documents (including documents prepared and implemented by the government, the MoHME, the parliament, related organisations, media, health insurance, as well as WHO and non-governmental organisation (NGO) reports, news sites and scholarly literature on HCV policy-making) were also consulted and thoroughly inspected. The interviewees were selected through purposive sampling and snowballing. Participants who had experience and knowledge about HCV-related programmes were interviewed and asked to introduce further actors involved with HCV-related policies. The interviews continued until a new actor was added and until data saturation was achieved. At the beginning of the interview, a consent form was given to the interviewees and they were informed about the aims of the present study. Interviewed participants included policy- and decision-makers, medical specialists, physicians, health department administrators, university professors, medical academicians, journalists, nurses, hospital and insurance managers, and pharmacists. Interviews were conducted by two authors (MB and HAG). Based on the views of the participants, data were analysed by three authors (MB, HAG and AR). Subsequently, after collecting data, a list of all actors and stakeholders was obtained based on both interviews and document searches. All documents related to HCV in the field of prevention and treatment, of any type (meeting abstracts and conference proceedings, laws, regulations, rules, reports, articles, books, news and speeches) were evaluated (Table 2).

**Table 2:** List of HCV-related documents consulted in the present study

Type of document	Number
Laws, regulations, rules	8
Guidelines, programmes	12
Websites	52
Reports	10
Scientific texts	7
Books	8

An ad hoc questionnaire was created in which stakeholders were rated in terms of interest, position, power and influence on HCV-related programmes and policies. Specifically, interest was defined as the degree of involvement with a given HCV-related policy; position was defined as the number of connections or interactions with other actors involved in the process of HCV-related policy-making; power was defined as the degree or extent to which the stakeholder was deemed likely to affect and modify a certain HCV-related policy; and influence was defined as the amount of potential benefits (including money, facilities, mobilised resources and knowledge) relevant to HCV-related decisions. For each item of the questionnaire (interest, position, power and influence) a five-point answer (low, low-medium, medium, medium-high, and high) was possible.

During the second phase, the list containing all the names of stakeholders/actors involved with HCV-related programmes and policies in Iran and the questionnaire were sent by email to the interviewees. Finally, the average score of the participants for each stakeholder was computed.

### Social network analysis (SNA)

A network characterised by properly balanced interactions among different stakeholders can lead to the development and implementation of adequate health-related policies [29]. Analysing this network can be a way to understand the structure of the process of policy-making and the relationships and interactions among the different stakeholders [30] in terms of relevance of each actor [31].

SNA is a sophisticated computational approach that shows the structure of the process of communication, relationships and interactions among the various stakeholders [32]. SNA shows also which parts of the network need more attention and dialogue, in order to ensure a proper healthcare policy-making process [33] and to achieve effective cooperation between different stakeholders [34]. SNA enables computing of network metrics, including degree, closeness, betweenness and eigenvector centralities.

Specifically, degree centrality is the number of direct relationships (links/edges connecting neighbouring nodes) that a stakeholder has [35]. Closeness centrality (or simply closeness) is the reciprocal of the sum of the length of the shortest interactions (paths, links or edges) between a stakeholder (node) and all the other actors (nodes) [36]. Betweenness centrality is based on the shortest paths, wherein a stakeholder can, indeed, reach other stakeholders (neighbouring nodes) through different paths, but only the shortest one is computed [37]. Eigenvector centrality is a proxy of the influence of a stakeholder and its importance in terms of connections with high-scoring (‘central’) nodes [38]. To carry out SNA, average scores were used based on the views of the participants, as well as the association of each organisation/institution with all the other organisations. Data were visualised and analysed using Gephi Version 0.92 software.

## Results

### Stakeholder analysis

The length of each interview varied between 10 and 15 min. After interviewing a sample of 18 participants and reviewing related and pertinent documents, 29 HCV-related stakeholders in Iran were identified, as reported in Table 3.

**Table 3:** List of stakeholders involved with HCV-related policies and programmes in Iran

Category	Stakeholder
Governmental (16 stakeholders)	Parliament (*Majlis*), Judicial system, State Prisons and Security and Corrective Measures Organisation, Ministry of Health and Medical Education, Imam Khomeini Relief Foundation, Law Enforcement Force, Government pharmaceutical companies, Government insurances, Medical Universities, Iranian Blood Transfusion Organisation, Ministry of Education, State Welfare Organisation, Military forces, Ministry of Sport and Youth, Ministry of Science, Research and Technology, Iran Drug Control Headquarters
NGOs (6 stakeholders)	Municipalities, non-governmental organisations, Scientific centres, Elites, Iranian Red Crescent, Clerics
Media (3 stakeholders)	Islamic Republic of Iran Broadcasting, news websites, social media
Private sector (2 stakeholders)	Private pharmaceutical companies, private insurances
International agencies (2 stakeholders)	WHO, Eastern Mediterranean Regional Organization

Specifically, identified stakeholders included the Iranian Parliament (*Majlis*, directly elected with the vote of the people, involved in legislation, approval and monitoring of funds, and enforcement of the laws), the Judicial system, the State Prisons and Security and Corrective Measures Organisation (SPSCMO), the MoHME, the Imam Khomeini Relief Foundation (IKRF, which sponsors and provides funding to poor people in the community), the Law Enforcement Force (LEF), governmental pharmaceutical companies (GPCs, involved in the preparation and production of medicines with the support of government funds), governmental insurances (GIs), MUs, the Iranian Blood Transfusion Organisation, the Ministry of Education (MoE), the State Welfare Organisation (SWO, involved in the improvement of social welfare, supporting unemployed people and developing ad hoc training programmes), the military forces, the Ministry of Sport and Youth (MoSY), the Ministry of Science, Research and Technology (MoSRT), the Iran Drug Control Headquarters (IDCH), the Municipalities, NGOs, scientific centres, the elites, the Iranian Red Crescent (IRC, which provides medicines and support to poor and ill people), the clerics, the Islamic Republic of Iran Broadcasting (IRIB, state-directed), news websites (society-directed), social media (society-directed), private pharmaceutical companies (PPCs, involved in the preparation and production of medicines with the financial support of the private sector), private insurances (PIs), WHO, and EMRO.

Stakeholders were classified into five groups, namely (1) governmental, (2) NGOs, (3) media, (4) members of the private sector, and (5) international agencies. More in detail, 16 stakeholders (55.17%) related to HCV-policies and programmes in Iran were governmental agencies. Six stakeholders (20.68%) were NGOs. Three stakeholders (10.34%) were political and media groups, whilst two stakeholders (1.73%) were members of the private sector and international agencies.

The identified stakeholders were involved in different steps of the process of HCV-related policy-making. For instance, the MoHME was involved in policy development, implementation and evaluation, whereas SPSCMO in implementation, MUs in policy implementation and evaluation, and IDCH in policy development. Estimated interest, position, power and influence of key stakeholders involved with HCV policy-making in Iran are shown in Table 4.

**Table 4:** Stakeholders involved with the process of HCV-related policy-making in Iran rated according to four items (interest, position, power and influence)

Stakeholders	Interest	Position	Power	Influence
Clerics	Low	Low	Low	Medium
Eastern Mediterranean Regional Organization	High	Low	Low	Low-medium
Elites	Medium	Low-medium	Low	High
Government insurances	Medium	Medium-high	High	High
Government pharmaceutical companies	Medium	Medium-high	Medium	High
Imam Khomeini Relief Foundation	Low-medium	Low	Low	Medium
Iran Drug Control Headquarters	Medium	Low	Medium-high	Medium
Iranian Blood Transfusion Organisation	High	High	Medium	High
Iranian Red Crescent	Low-medium	Low	Low	Medium
Islamic Republic of Iran Broadcasting	Low-medium	Low	Low-medium	High
Judicial system	Low-medium	Low	Medium-high	Medium
Law Enforcement Force	Low-medium	Low	Low	Low-medium
Medical Universities	High	High	Medium-high	High
Military forces	Low	Low	Low	Medium
Ministry of Education	Low	Low	Low	Medium
Ministry of Health and Medical Education	High	High	High	High
Ministry of Science, Research and Technology	Low	Low	Low	Low
Ministry of Sport and Youth	Low	Low	Low	Medium
Municipalities	Low	Low-medium	Low	Low
News websites	Medium	Low	Medium	Medium
Non-governmental organisations	Low-medium	Low	Low	Medium
Parliament	Low-medium	Low	High	High
Private insurances	Medium	Low	Low	Medium-high
Private pharmaceutical companies	Medium-high	Low	Low	Medium
Scientific centres	Medium	Low	Low	Medium-high
Social media	Medium	Medium	Medium	Medium
State Prisons and Security and Corrective Measures Organisation	Medium-high	High	High	Medium-high
State Welfare Organisation	Low-medium	Medium	Medium	High
WHO	High	Low	Medium	Medium

### Interest analysis

Among the stakeholders identified, the MoHME and the MUs (the main healthcare providers in Iran), the IBTO (the major provider of blood transfusion and blood donation), WHO and EMRO had the highest interest level towards HCV-related policies in Iran. Moreover, according to the scores given by the participants, the SPSCMO and PPCs had a medium-high interest for HCV-related policies. Prisoners, as one of the most high-risk groups for HCV, have led the SPSCMO authorities to take an interest in harm reduction programmes for prisoners. PPCs have become interested in developing anti-HCV drugs in recent years. Elites, GIs, GPCs, IDCH, news websites, PIs, scientific centres and social media had medium interest for the process of HCV-related policy-making. Stakeholders like the IKRF, the IRC, the IRIB, the Judicial system, the LEF, NGOs, the Iranian Parliament, and SWO were found to have a low–medium interest for HCV-related policy-making. Other stakeholders, such as clerics, municipalities, military forces, the MoE, the MoSRT, and the MoSY, had a low interest towards HCV-related policies. Thus, international agencies had a high interest for HCV-related policy-making, whereas media and members of the private sector were characterised by a medium interest and governmental bodies and NGOs by a highly variable interest, ranging from low to high, depending on the specific organism.

### Position analysis

The MoHME has been recognised as the most important stakeholder in terms of position, having much support for all its activities aimed at preventing, controlling and treating the disease. Additionally, stakeholders such as the IBTO, MUs and the SPSCMO were found to rank high in terms of position. Furthermore, GIs and GPCs were rated medium–high; they have, indeed, increased their support for reducing the costs and helping patients to adhere to treatment. Social media and SWO were rated medium, whereas municipalities and elites were rated low–medium in terms of position. PPCs also play a relatively important role in patient treatment programmes, despite being rated low together with clerics, the EMRO, the IKRF, the IDCH, the IRC, the IRIB, the LEF, the Judicial system, the military forces, the MoE, the MoSRT, the MoSY, news websites, NGOs, the Iranian Parliament, PIs, scientific centres, and WHO. Summarising, media as well as members of the private sector, NGOs and international agencies were rated low in terms of position, were governmental actors were rated low to high.

### Power analysis

Participants rated the Parliament, the MoHME, GIs, and the SPSCMO as the most important HCV-related policy- and decision-makers in terms of power. In recent years, for instance, the SPSCMO has taken effective steps to control the disease through screening activities for prisoners in collaboration with the MoHME and the Judicial system. The latter, together with IDCH and the MUs, has a medium–high power. Actors like GPCs, the IBTO, news websites, social media, SWO, and WHO were found to have medium power, whereas the IRIB was rated low–medium. In general, most stakeholders, including clerics, the EMRO, elites, the IKRF, the IRC, LEF, military forces, the MoE, the MoSRT, the MoSY, municipalities, NGOs, PIs, PPCs, and scientific centres, were rated low in terms of power. Many organisations, despite having the institutional tasks and duties of controlling the spread of HCV, do not use their power to influence HCV-related plans and programmes in Iran. In summary, media were rated medium in terms of power, whereas international agencies and members of the private sector were respectively rated low to medium and low. Non-governmental actors were generally rated low, whilst governmental bodies were rated low to high.

### Influence analysis

The MoHME, the Iranian Parliament, GPPs, GIs, MUs, the SWO, the IRIB, and the IBTO have a great influence on the policy of HCV-related screening and treatment programmes, as well as on the economic and financial plans. In general, the community elites have an important role to play in influencing policies, and they can provide Iran with more effective programmes by communicating effectively with policy- and decision-makers and expressing their views. PIs, scientific centres, and the SPSCMO were rated medium–high whereas clerics, the IKRF, the IDCH, the IRC, the Judicial system, the military forces, the MoE, the MoSY, news websites, NGOs, PPCs, social media and WHO were rated medium. EMRO and LEF were considered to have a low–medium influence, whereas the remainder (the MoSRT and municipalities) were judged to have a low influence. Summarising, media, members of the private sectors and international agencies were rated medium in terms of influence, whereas non-governmental and governmental actors were respectively rated low to medium and low to high.

### Social network analysis (SNA)

**Fig. 1:**
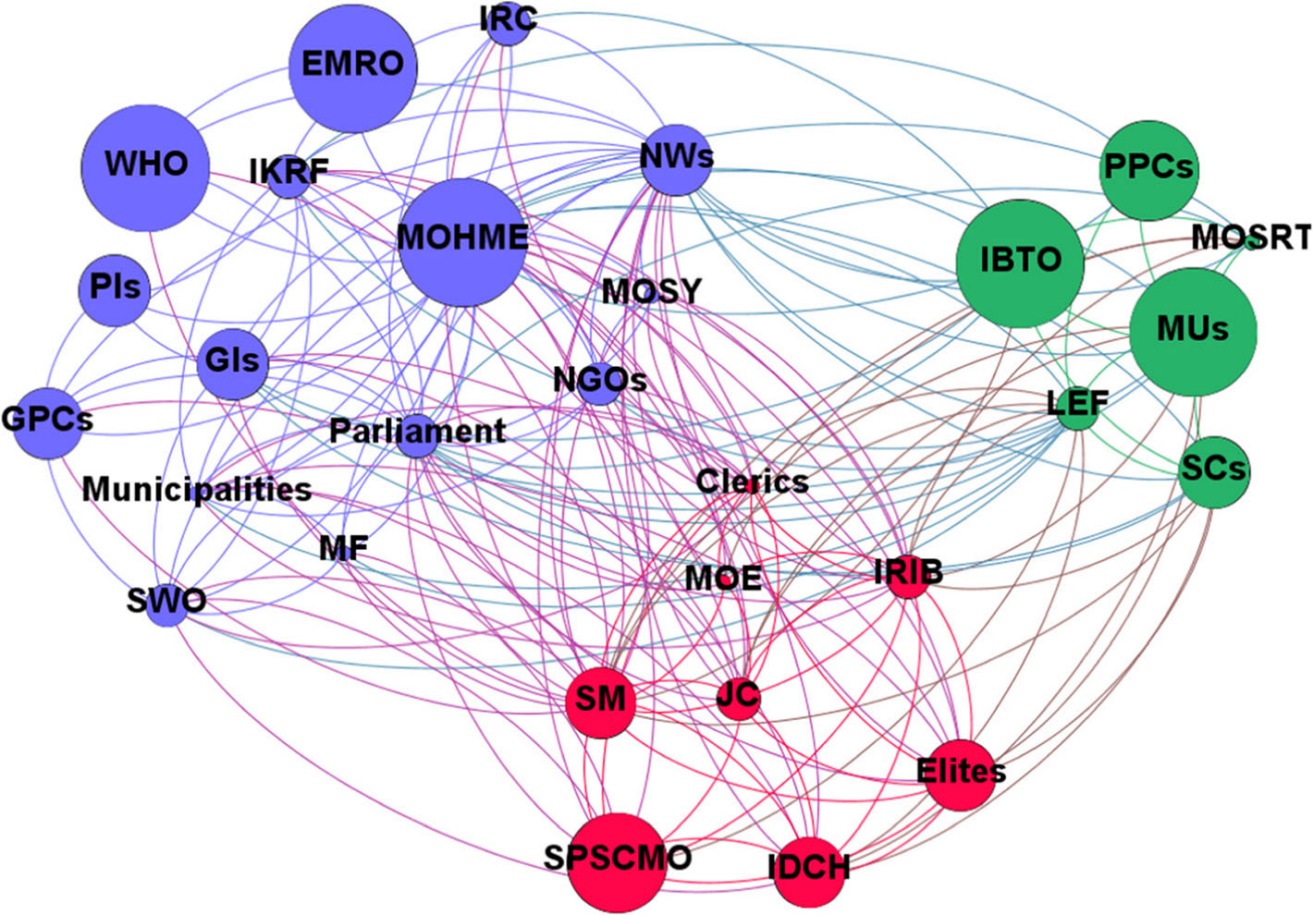
Network map based on the interest of different stakeholders involved with HCV policy-making in Iran

**Fig. 2:**
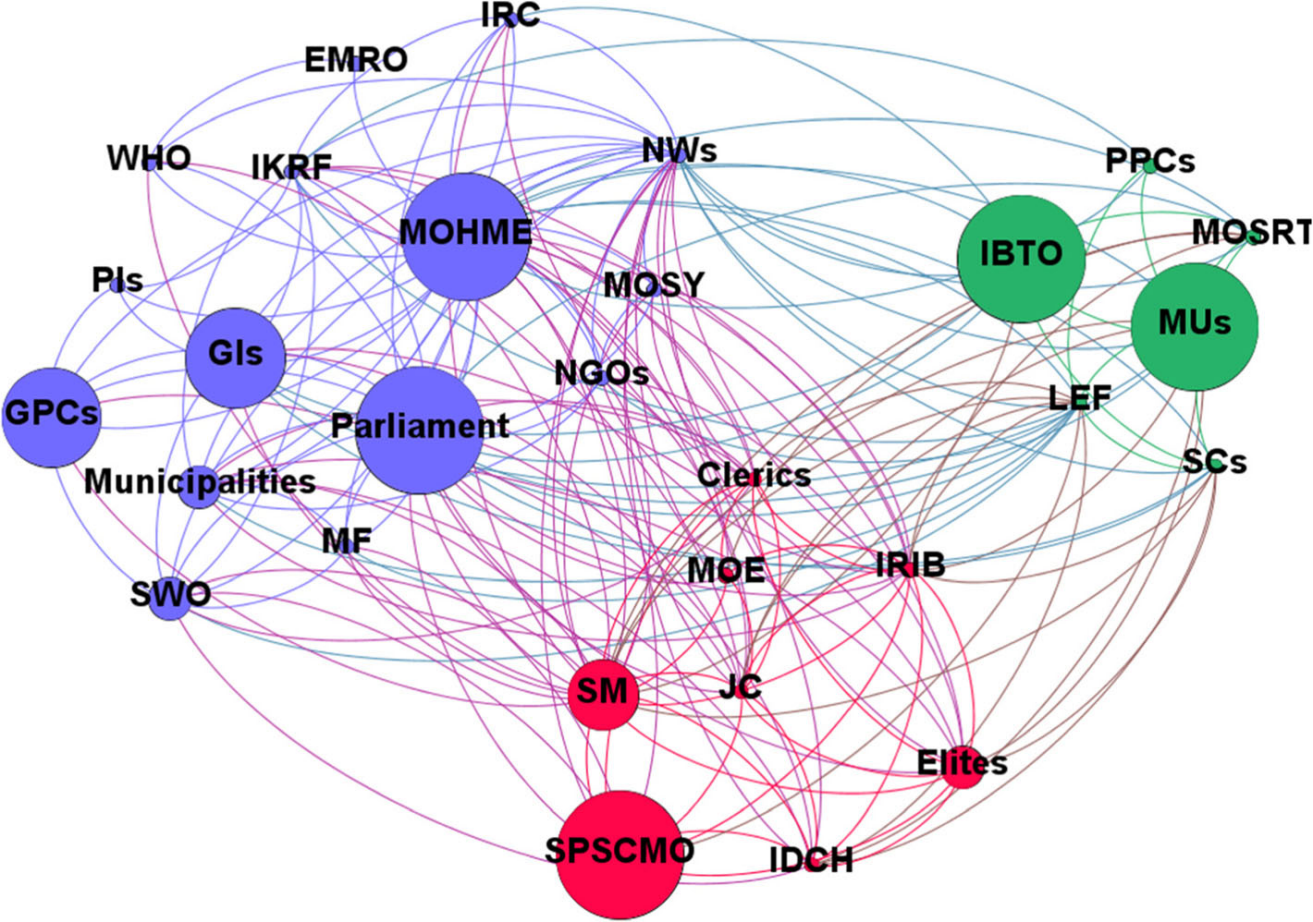
Network map based on the position of different stakeholders involved with HCV policy-making in Iran

**Fig. 3:**
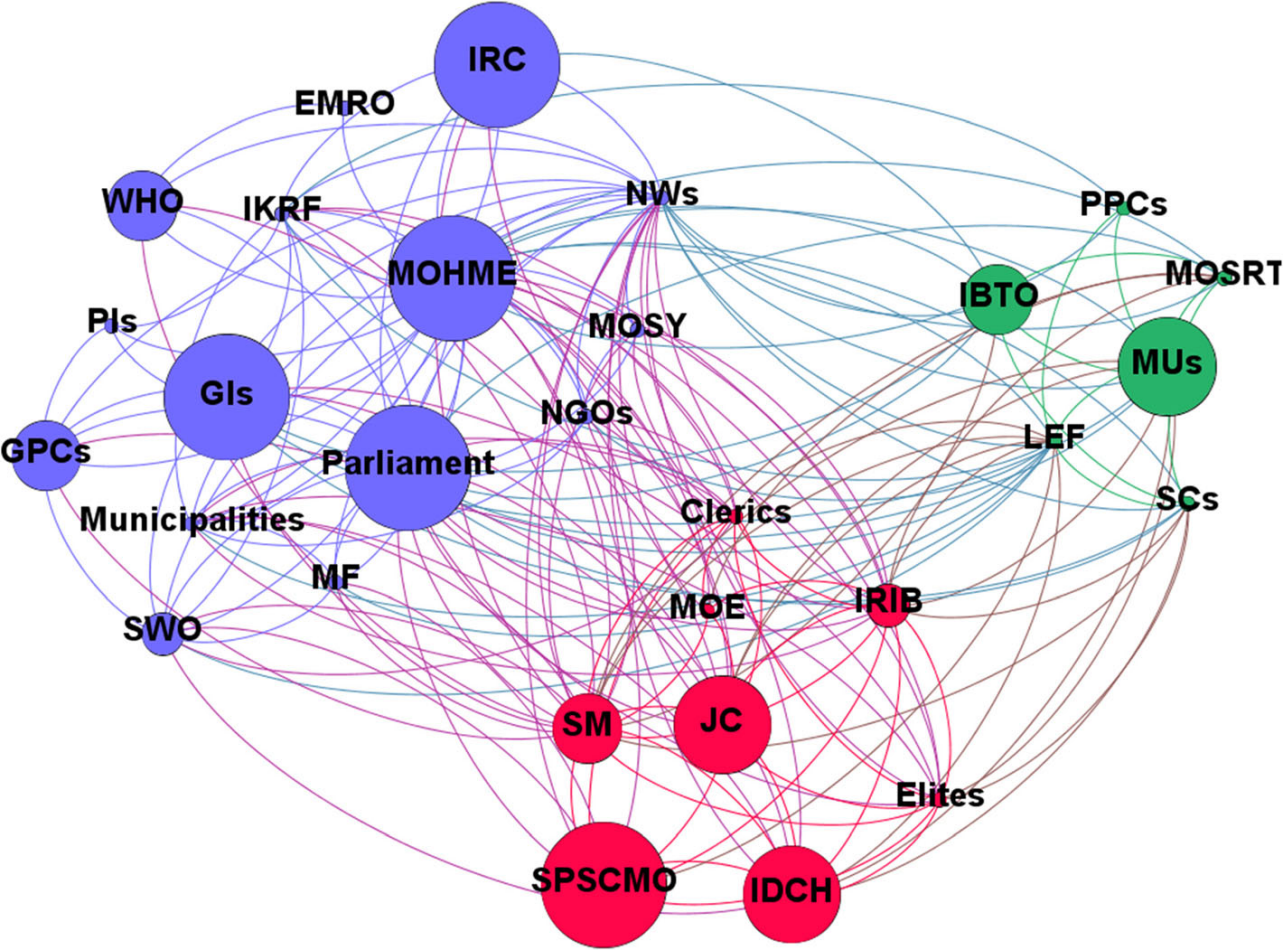
Network map based on power of different stakeholders involved with HCV policy-making in Iran

**Fig. 4:**
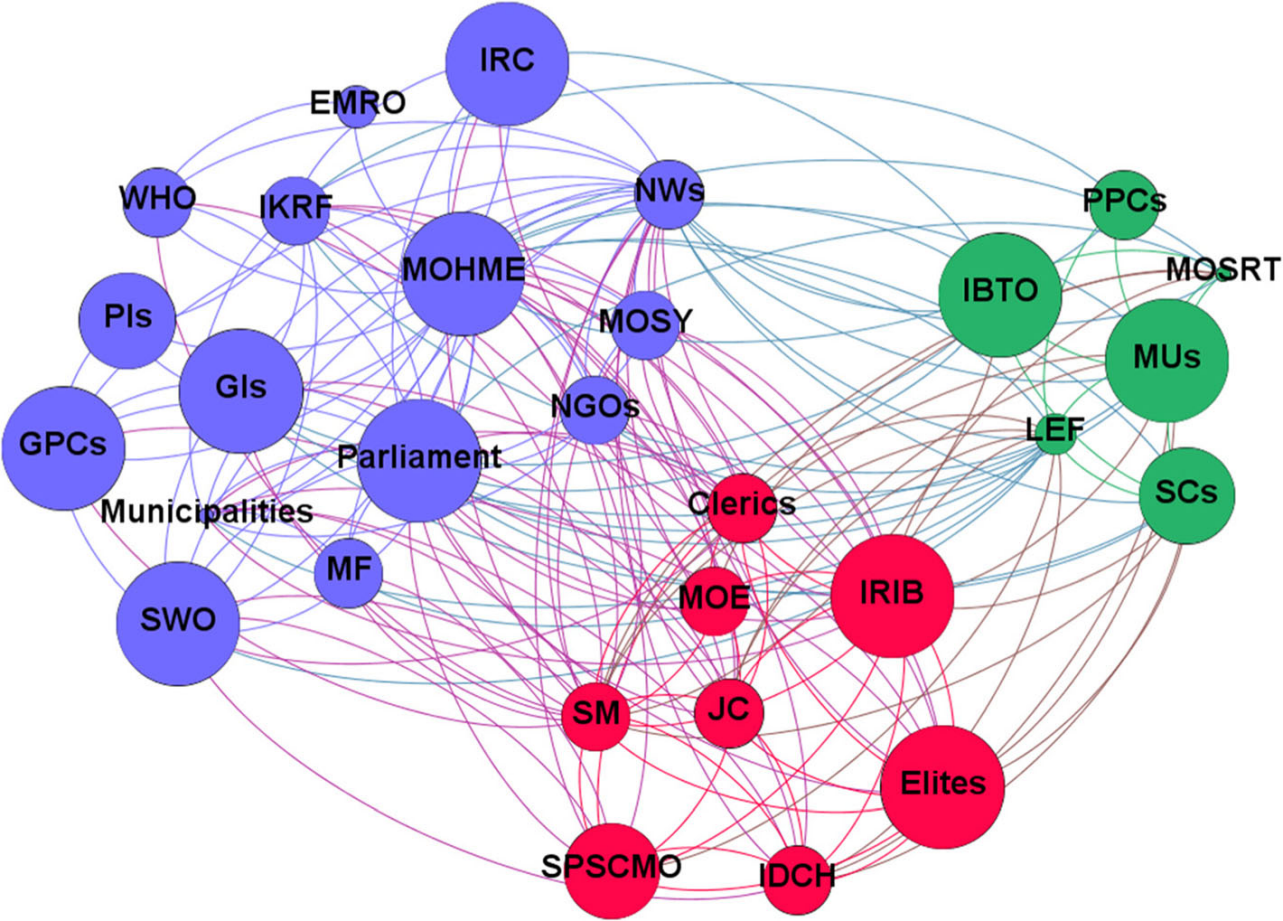
Network map based on influence of different stakeholders involved with HCV policy-making in Iran

The computed network metrics, including degree, closeness, betweenness and eigenvector centralities, are reported in Tables 5 and 6. Based on the SNA-related items, the MoHME had the highest rank for HCV-related policies in Iran, being the authority which writes, enforces and funds healthcare policies. Based on these findings, stakeholders like social media, news websites and the IRIB are the most centralised after the MoHME and Parliament. Network and node-level metrics are shown in Tables 5 and 6. Network maps based on interest, position, power and influence of the different stakeholders involved with HCV policy-making in Iran are respectively shown in Figs. 1, 2, 3 and 4.

**Table 5:** Network metrics, including degree, closeness, betweenness and eigenvector centralities, computed for the different stakeholders involved with HCV policy-making in Iran

Stakeholders	Degree centrality	Closeness centrality	Betweenness centrality	Eigenvector centrality
Ministry of Health and Medical Education	29	1	74.85	1.00
Parliament	25	0.90	26.18	0.97
Social media	25	0.90	26.18	0.97
News websites	25	0.90	26.18	0.97
Islamic Republic of Iran Broadcasting	18	0.73	7.44	0.78
Law Enforcement Force	17	0.71	9.01	0.73
Judicial system	16	0.70	6.50	0.70
Clerics	15	0.68	4.69	0.67
Elites	14	0.66	2.64	0.66
Imam Khomeini Relief Foundation	14	0.66	9.58	0.59
Municipalities	13	0.65	2.49	0.61
Government insurances	13	0.65	6.08	0.55
Medical Universities	13	0.65	5.41	0.58
Iran Drug Control Headquarters	13	0.65	1.69	0.63
State Prisons and Security and Corrective Measures Organisation	12	0.63	1.23	0.59
State Welfare Organisation	12	0.63	2.43	0.55
Scientific centres	11	0.62	1.60	0.53
Non-governmental organisations	11	0.62	1.11	0.54
Ministry of Science, Research and Technology	9	0.59	0.74	0.43
Iranian Blood Transfusion Organisation	9	0.59	1.00	0.41
Military forces	8	0.58	0.21	0.41
Iranian Red Crescent	8	0.58	0.56	0.40
Government pharmaceutical companies	8	0.58	1.43	0.38
Ministry of Education	8	0.58	0.30	0.42
Ministry of Sport and Youth	7	0.57	0.09	0.38
WHO	6	0.56	2.00	0.30
Private insurances	4	0.53	0.14	0.16
Private pharmaceutical companies	4	0.53	0.12	0.19
Eastern Mediterranean Regional Organization	2	0.51	0.00	0.08

**Table 6:** Network and node-level metrics

Parameter	Value
Nodes	29
Edges	184
Density	0.453
Average degree	12.69
Average clustering coefficient	0.746
Number of triangles	564
Diameter	2
Average path length	1.546

## Discussion

The findings of this study showed that the identified stakeholders have a different spectrum of interest, position, power and influence on HCV-related policies in Iran. Identifying these stakeholders is an important factor for succeeding in developing and implementing effective interventions and mobilising all necessary resources and facilities [39, 40]. Regarding HCV-related policy in Iran, the identified stakeholders directly and indirectly influence the formulation and implementation of policies. According to participant’s viewpoints, the MoHME is the most important stakeholder identified.

Disagreements and different standpoints concerning the development and implementation of healthcare policies can be a challenge [41], despite negotiations being able to mitigate such contrasts and contribute to finding an optimal compromise [42]. In the case of HCV-related policies in Iran, a lack of cooperation has hindered a proper implementation of the plans [10]. Based on the ranks and metrics properties of the stakeholders, one of the noticeable findings of the present investigation is that, despite the fact that HCV has been properly identified as a challenge for Iran’s health sector, many stakeholders believe that they could not do much. Indeed, many stakeholders tend to consider the disease merely as a priority of the MoHME.

The identified stakeholders showed different degrees of interest towards HCV-related policy. Their differing motivation can explain this [43]; for example, interest by the IKRF and SWO was found to be low despite being important organisations that help poor people. On the other hand, stakeholders rated high for position can have a high impact on other stakeholders. The political, economic, social and cultural status of each stakeholder indeed plays an important role in the process of policy-making. The power of stakeholders is one of the main factors and components of the development and implementation of health-related policies [44]. However, regarding HCV-related policy in Iran, participation and involvement in political discussions for policy-making are generally low, and this is a challenge that can affect policy implementation by causing delays or resulting in failures.

Experts believe that, in order to improve policy and implementation, both top-down and bottom-up approaches should be used to allow continuous implementation of policies [45]. Specifically with regards to HCV policy in Iran, most decisions are taken by the MoHME, with a low participation of other stakeholders and a lack of appropriate dialogue with the bottom-up beneficiaries. Through a more intense political dialogue, these weaknesses could be somewhat reduced. Moreover, due to the limited financial resources and economic difficulties caused by international sanctions in Iran, not all the necessary financial resources for the treatment of patients are provided to every patient. Although some new financial resources have been planned since the implementation of the Health Transformation Plan in Iran, these resources have not been fully allocated [46].

Stakeholders can influence policies based on their own characteristics and their effects can be positive or negative [47]. Regarding the prevention dimension, IRIB, social media, news websites and clerics have a great influence on society. Unfortunately, despite this high potential, because of certain cultural problems associated with HCV, such as stigma, they have not been successful in fulfilling the mission of raising awareness. However, in recent years, the MoHME has tried to reduce HCV-related stigma, and stakeholders are working with the MoHME to raise awareness among the general population.

The findings of the present study showed that network density was generally low. Collaboration and interactions among all involved stakeholders at all levels should be extensively adopted to address important issues such as HCV. They should have a comprehensive understanding of the magnitude and impact of their collaboration.

Cultural factors and community views can affect health policies [48, 49]. In the Iranian society, stigma is still high and, therefore, SNA findings showed that those who are responsible for spreading information and raising awareness should have a more active participation and a greater role in preventive policies; the centrality of stakeholders such as social media, news websites and the IRIB emphasised this issue.

Finally, the findings of this study showed that the ongoing dialogue between stakeholders favours more sensitivity and empowerment, potentially resulting in an integration of some programmes. Consultation with all involved stakeholders can help in the design and implementation of adequate policies.

## Conclusion

The findings of this study showed that there are many stakeholders involved with HCV-related policy in Iran. The extent of their participation and support in the process of policy-making is different and variable. In general, involvement in HCV-related policy-making is low. This information has practical implications for policy- and decision-makers, helping them to adopt more favourable and effective strategies. HCV-related policy-making and decision processes should not be handled by the MoHME alone, but should receive support and advocacy from all involved stakeholders. This is essential for succeeding in the ambitious goal of managing and controlling this disease. Proper policy-making involves trust, ongoing negotiations, integration and implementation of the different views of all involved stakeholders. In the present investigation, SNA was a critical point for identifying stakeholders as well as gaps and weaknesses so that policy- and decision-makers can have a more comprehensive understanding of health issues and processes and make better-informed decisions.

## Abbreviations

EMRO: Eastern Mediterranean Regional Organization
GIs: government insurances
GPC: government pharmaceutical companies
HCV: hepatitis C virus
IDCH: Iran Drug Control Headquarters
IKRF: Imam Khomeini Relief Foundation
IRC: Iranian Red Crescent
IRIB: Islamic Republic of Iran Broadcasting
LEF: Law Enforcement Force
MoE: Ministry of Education
MoHME: Ministry of Health and Medical Education
MoSRT: Ministry of Science, Research and Technology
MoSY: Ministry of Sport and Youth
MUs: medical universities
NGOs: non-governmental organisations
PIs: private insurances
PPCs: private pharmaceutical companies
SNA: social network analysis
SPSCMO: State Prisons and Security and Corrective Measures Organisation
SWO: State Welfare Organisation
